# Cost-minimization analysis of the direct costs of TPE and IVIg in the treatment of Guillain-Barré syndrome

**DOI:** 10.1186/1472-6963-11-101

**Published:** 2011-05-16

**Authors:** Jeffrey L Winters, David Brown, Elisabeth Hazard, Ashok Chainani, Chester Andrzejewski

**Affiliations:** 1 Department of Laboratory Medicine and Pathology, Mayo Clinic, 200 First ST SW, Rochester, MN 55905, USA; 2 Pharmacy Department, Health New England, One Monarch Place STE 1500, Springfield, MA 01144, USA; 3 Pricing & Reimbursement Strategy, ACE Strategic Reimbursement, 909 Jessica Terrace, Downingtown, PA 19335, USA; 4 Pathology Department, Baystate Medical Center, 759 Chestnut ST, Springfield, MA 01199, USA

**Keywords:** plasma exchange, intravenous immunoglobulin, Guillain-Barré syndrome, cost effectiveness

## Abstract

**Background:**

Controlled trials have found therapeutic plasma exchange (TPE) and intravenous immunoglobulin (IVIg) infusion therapy to be equally efficacious in treating Guillain-Barré syndrome (GBS). Due to increases in the price of IVIg compared to human serum albumin (HSA), used as a replacement fluid in TPE, we examined direct hospital-level expenditures for TPE and IVIg for meaningful cost-differences between these treatments.

**Methods:**

Using financial data from our two institutions, hospital cost profiles for IVIg and 5% albumin were established. Reimbursement amounts were obtained from publicly available Medicare data resources to determine payment rates for TPE, non-tunneled central catheter line placement, and drug infusion therapy. A model was developed which allows hospitals to input cost and reimbursement amounts for both IVIg and TPE with HSA that results in real-time valuations of these interventions.

**Results:**

The direct cost of five IVIg infusion sessions totaling 2.0 grams per kilogram (g/kg) body weight was $10,329.85 compared to a series of five TPE procedures, which had direct costs of $4,638.16.

**Conclusions:**

In GBS patients, direct costs of IVIg therapy are more than twice that of TPE. Given equivalent efficacy and similar severity and frequencies of adverse events, TPE appears to be a less expensive first-line therapy option for treatment of patients with GBS.

## Background

Guillain-Barré syndrome (GBS), the most common cause of acute neuromuscular paralysis in the U.S. with an incidence of 1 to 2 per 100,000, is an immune mediated demyelinating polyneuropathy mainly affecting motor and sensory peripheral nerves. Characterized by paresthesias, weakness, and ascending paralysis in a distal to proximal pattern, GBS patients may also demonstrate autonomic nerve dysfunction further complicating their recovery. The majority of severely affected patients require hospitalization, especially those with oropharyngeal and respiratory muscle involvement. Ventilator support may be needed in 30% of all GBS patients [1].

Efficacy of therapeutic plasma exchange (TPE) has been demonstrated in randomized trials comparing supportive care or corticosteroid therapy in GBS patients. TPE reduces ventilator support days and shortens time to unaided walking resulting in earlier hospital discharge [2]. Trials comparing TPE with intravenous immunoglobulin (IVIg) have demonstrated equivalency of the two in shortening time to unaided walking and reducing length of ventilator support [3]. In a summary of five trials with a combined enrollment of 582 patients, TPE when compared to IVIg was found to be equivalent with regard to improvement in disability grade with no significant differences in other outcome measures [4]. Based on such data demonstrating equivalence of these two treatment options, the American Academy of Neurology has concluded that TPE and IVIg are equivalent and recommended either for the treatment of non-ambulatory patients [5].

While TPE and IVIg are equally effective in the treatment of GBS, concern has arisen over whether the safety profile of the two treatments is equivalent. The initial trial comparing IVIg to TPE found a lower overall adverse event rate with IVIg [6]. A subsequent larger trial, however, found similar adverse event rates, with the majority being transient and mild [3]. Subsequent meta-analysis of available data found a relative risk of complications with IVIg compared to TPE of 0.84 but the 95% confidence interval (0.54 to 1.30) crossed 1, indicating equivalency [7]. These findings suggest that not only are the two treatments equivalent with regard to patient response but also with regard to potential risks.

Many physicians, however, prefer IVIg for the treatment of patients with severe GBS. This preference may be due partly to the 1997 Plasma Exchange/Sandoglobulin Guillain-Barré Syndrome Trial that reported, "On grounds of equal therapeutic benefit, greater convenience and similar overall cost, IVIg may be preferable to TPE for treatment of adult patients with Guillain-Barré syndrome...provided there are no contraindications to IVIg" [3]. At the time of this trial, the average price of IVIg in the U.S. was approximately $30 per gram [8]. In the decade following this study, prices for IVIg increased to >$60 per gram for liquid formulations and >$50 per gram for the powder formulation [8]. Additionally, in 2005, manufacturers instituted allocation programs due to supply shortages that restricted IVIg availability. Some hospitals have had to establish triage plans allocating IVIg use only for approved indications whereas others have required consideration of alternate treatments, like TPE, for patients with neurologic disorders [9]. Several factors contribute to IVIg shortages including consolidation in the blood product industry, the switch from the manufacturing of powder to liquid preparations, production cuts by some manufacturers and a lag time in production when firms change manufacturing processes [10]. Additional supply pressures result from steadily increasing clinical demands for the off-label use of IVIg at a rate of 5-10% annually [11]. During these periods, IVIg prices have risen more than 20% and as of April 2010, the average price for liquid formulations of IVIg had increased to $70.22 per gm.

During this same time, human serum albumin (HSA) prices averaged $30 for a 250 ml bottle. HSA prices reached a low in 2004-2005 and were less than one-half the1999 price in 2007. As of April 2010, the price of a 250 ml bottle of 5% HSA was $34.06.

Based on the above considerations, we re-examined the assumption of cost parity between a standard course of IVIg and a series of five TPEs by performing a cost-minimization analysis to determine whether meaningful differences in the direct cost currently exist between these two therapeutic options.

## Methods

A cost-minimization analysis comparing a typical course of five TPEs against five IVIg infusions was performed. The model was developed from the study data included in Tables 1 and 2 and was created in a spreadsheet (Excel 2003, Microsoft Corporation, Redmond WA) and is available as additional file 1 to this manuscript. The model provides hospitals an opportunity to include their own direct costs and reimbursement amounts from any specific payer to arrive at real-time values.

**Table 1:** Direct hospital costs of a five-treatment inpatient course of intravenous immunoglobulin (IVIg)

Resource	Cost *
IVIg, 0.4 g/kg (70 kg patient) × 5 infusions @ $70.22/g^a^	$9,830.80
Direct RN labor cost (73 min × $0.79/min)^b ^cost × 5 infusions	$474.00
IVIg infusion supplies × 5 infusions^c^	$25.05
TOTAL COST	$10,325.05

^a ^IVIg hospital cost $70.22 shown above is based on hospital contracted prices paid to manufacturers. Starting in Q1-2011 Medicare OPPS (outpatient prospective payment system) IVIg reimbursement is $73.30/gram based on average of five IVIg brands. Medicare payment rates for Q1-2011 are based on 106% of third quarter 2010 manufacturer's reported average sales price, which serves as a proxy for hospital acquisition cost, distributor mark-up and direct overhead including storage, preparation and disposal costs).

^b ^Center for Medicare and Medicaid Services (CMS). CY 2010 Labor file. RUC source for CPT 96365 and 96366. Assumes an average two-hour infusion (one unit each of CPT 96365 + CPT 96366), which utilizes 59 minutes and 14 minutes (total 73 minutes) of nurse clinical labor time. This value understates typical labor costs for a PE nurse operator, which is better approximated by the RUC value of $0.79/minute applied for therapeutic intravenous infusions; we used that higher labor rate in our cost model.

^c ^CMS. CY 2010 Supplies file. RUC source for CPT 96365 and 96366.

**Table 2:** Direct hospital costs of a five-treatment inpatient course of therapeutic plasma exchange (TPE)

Resource	Cost *
Tubing set and all other supplies ($210.08)^a ^× 5 procedures	$1,050.40
Direct RN labor cost (120 min × $0.79/min)^b ^cost × 5 procedures	$474.00
Insert central venous catheter (incl. catheter kit, supplies, x-rays); hospital costs^c^	$750.81
5% albumin × 5 procedures^d^	$1,990.00
TPE equipment amortization^e ^× 5 procedures ($52.59/procedure)	$262.95
Service contract amortization^f ^× 5 procedures	$110
TOTAL COST	$4,638.16

^a ^CMS. CY 2010 Supplies file. PEAC (Practice Expense Advisory Committee) source for CPT 36514. March 2004 update.

^b ^CMS. CY 2010 Labor file. PEAC source for CPT 36514. March 2004 update. For PE (CPT 36514), the CMS Labor file cites a labor cost of $0.42/minute, based on an RN/LVN operator. This value understates typical labor costs for a PE nurse operator, which is better approximated by the RUC value of $0.79/minute applied for therapeutic intravenous infusions; we used that higher labor rate in our cost model.

^c ^CMS. CY 2010 payment amount for APC 0621. (Medicare-reported true median hospital cost for insertion of non-tunneled central venous catheter or PICC line; paid under APC 0621; surgeon's fee is separately paid.)

^d ^40 ml/kg 5% albumin (50 ml/kg total fluid volume replacement with 80% albumin:20% saline) × 70 kg patient × 5 procedures = 14,000 ml = 56 vials of 250 ml 5% albumin × $35.53/vial = $1,990 Albumin cost of $35.53 per vial represents the January 2011 provider contracted acquisition cost. Medicare payment rate is set at 95% of AWP for albumin, which serves as a proxy for hospital acquisition cost, distributor mark-up and direct overhead including storage, preparation and disposal costs.

^e ^CMS. CPEP equipment file, March 2004 update (most recent data). Includes cell separator system, blood warmer and medical recliner chair, with useful lives of 6, 7 and 10 years (200 procedures per year) and costs of $59,320, $3,840 and $829, respectively.

^f ^$4,450 annually per cell separator system (CaridianBCT device); assumes 200 procedures per year per device.

### Assumptions

Basic assumptions about each treatment regimen are given in Table 3. With regard to the number of TPE procedures, in patients not requiring respiratory support, a course of two TPEs has been compared to four with the later being superior in multiple outcome measures. In more severely affected individuals, a course of four TPEs was compared to six and both were found to be equivalent with the exception of a higher frequency of hypotension with six TPEs [12]. Based upon these findings, recommendations in published guidelines [13], and to maximize the costs of TPE, a course of five TPEs was selected. We conservatively selected the highest plasma exchange volume for this clinical indication based upon the medical literature [13]. We assumed an average 70 kg adult and an 80:20 replacement fluid mix of 5% HSA and normal saline, resulting in 14,000 ml of HSA to complete all five TPE procedures. Alternate replacement colloids to 5% HSA, such as hydroxyethyl starch solutions, can be used and are less expensive than 5% HSA. In addition, a lower percentage of HSA, such as 60:40 or 50:50 HSA to saline could also be used and would further reduce the costs of TPE. However, these alternate replacement fluids and fluid mixtures are associated with a higher frequency of reactions and are not consistent with recommendations for replacement fluids in published guidelines [13]. In addition, utilizing this replacement fluid mixture maximize the costs associated with TPE and represents a worst-case scenario. Central venous catheter placement for venous access in GBS patients treated with PE is not always necessary but we conservatively assumed that all patients would require this. We assumed that serious adverse events (AEs) attributable to TPE or IVIg occur infrequently at similar rates and that associated costs would not directly affect this analysis. Finally, we also assumed that the length of hospitalization would be equivalent between the two therapies given the equivalent time to response between the two [3,4]. Both therapies can be performed as an outpatient once the patient has responded.

**Table 3:** Basic assumptions applied regarding therapeutic plasma exchange and intravenous immunoglobulin infusion treatment regimens in study

Therapeutic Plasma Exchange (TPE)	Intravenous immunoglobulin infusion (IVIg)
• Five plasma exchanges totaling 250 ml/kg total plasma volume replacement • 80% volume replacement with 5% human albumin; 20% volume with saline or other crystalloid • 70 kg mean body weight • Non-tunneled subclavian or intrajugular catheter placed in all patients	• 5 IVIg infusions totaling 2.0 g/kg • 70 kg mean body weight • Infusions started and monitored by a nurse

### Sources of direct costs

Direct labor costs were obtained from the Center for Medicare and Medicaid Services (CMS) Clinical Practice Expense Panel (CPEP) clinical labor database for plasma exchange and intravenous infusion [14]. Supply costs for a TPE procedure, totaling just over $210, were identified in the CPEP supply database. The administration set and other supplies for an IVIg infusion cost less than $6 [14].

The direct hospital-level costs for HSA and IVIg are not published as prices and are driven by hospital contracts. CMS does, however, publish drug pricing files based on manufacturer reported average sales prices (ASP). Effective January 1, 2011 the agency uses these to develop reimbursement amounts by adding 6% to the manufacturer reported ASP for physician offices and the same 6% under outpatient prospective payment system (OPPS) for hospital outpatient reimbursement. Values used for reimbursement in this cost minimization analysis represent 106% of third quarter 2010 manufacturers' U.S. average sales prices for IVIg. For data used in the Budget Impact Model, we used the most current reimbursement rate for IVIg reimbursement from Medicare; the IVIg reimbursement of $73.226/gram equates with first quarter 2011 Medicare OPPS (outpatient prospective payment system) payment rate averaged across five liquid IVIg products (J1459, J1561, J1568, J1569, J1572) (Medicare reimbursement rates are for third quarter 2011 based on 106% of third quarter 2010 manufacturer's reported average sales price, which serves as a proxy for hospital acquisition cost, distributor mark-up and direct overhead including storage, preparation and disposal costs) [15].

HSA, however, is still reimbursed under the AWP (Average Wholesale Price) formula. These values serve as proxies for the hospital reimbursement and include acquisition cost, distributor mark-up, and direct overhead related to product storage, preparation and disposal [15]. For hospital drug costs we used average prices paid by the two hospitals, based on contracts. The average hospital cost from two hospitals for IVIg was $ 70.22/gram and the average cost for 5% HSA was $35.35/250 ml bottle.

The surgeon's fee for placement of a central venous catheter is not a hospital cost and is not included in the analysis. However, for those interested in considering a global direct cost comparison, the average Medicare surgeon payment for non-tunneled central venous line placement is $130. Physician medical management and oversight fees for either TPE or IVIg infusions were not included in the analysis.

## Results

Direct hospital costs related to IVIg and TPE treatment are provided in Tables 1 and 2. The estimated direct cost for IVIg infusion therapy to treat a 70 kg adult is $10,305 (Table 1). This is approximately 159% more than the $3,980 direct cost of a course of TPE for the same 70 kg adult (Table 2).

Whereas IVIg prices have risen in recent years, in contrast, HSA prices have been more volatile. At its cost peak in 1999-2000, the price for HSA at our two institutions averaged between $55 and $58 per 250 ml bottle. As HSA represents the single largest TPE cost element, we calculated a 50% and 100% increase in the current cost of $36.48 per 250 ml bottle of HSA to determine how this might affect the comparative cost outcome. As of January 1, 2011, CMS reimburses $67.07 per 250 ml bottle based on the AWP formula. Hospital costs for HSA are significantly lower than this amount, $35.53 for 250 ml. The price of HSA would have to increase approximately five fold for TPE to match the cost of IVIg drug costs.

## Discussion

Given the higher cost of IVIg relative to TPE, a reassessment of their clinical benefits and associated AEs is relevant. The Guillain-Barré Syndrome Study Group first established the efficacy of TPE when 245 patients were randomized to receive five to six one plasma volume exchanges over 14 days or "best supportive care" at 21 North American treatment sites [16]. In this study, the TPE group experienced a shorter median time to improve one grade (19 vs. 40 days; p < 0.001), better percentage of subjects who had improved one grade at 4 weeks (59% vs. 39%, p < 0.01), shorter time to walk unassisted (53 vs. 85 days; p < 0.001) and shorter mean time on ventilator support (24 vs. 48 days, p < 0.01). TPE and supportive care demonstrated no difference in complication frequency [16]. A Cochrane Database review of six clinical trials (649 patients) confirmed that TPE is superior to supportive care across multiple functional outcome measures [17]. TPE was associated with fewer severe sequelae after one year, fewer infectious events and fewer cardiac arrhythmias than supportive care. The reviewers concluded that TPE is "the first and only treatment that has been proven to be superior to supportive treatment alone," and should be regarded as the standard against which new treatments should be judged [17].

Consequently, TPE has served as the standard treatment arm for two large randomized trials examining IVIg use in patients with severe GBS. In 1992, the Dutch Guillain-Barré Study Group randomized 147 patients to five to six TPEs or five daily IVIg infusions of 0.4 grams/kg [6]. The use of IVIg was associated with a shorter median time to improve one grade and a significantly higher percentage of subjects who improved one or more grades at four weeks. At three-month examination, however, no significant difference in this primary outcome was seen [6]. Of note in this study those treated with TPE did no better than patients in the Guillain-Barré Syndrome Study Group trial who received supportive care. It was subsequently identified that the Dutch Guillain-Barré Study Group study did not appropriately compare the two therapies [18,19]. The definition of functional grade 3 as "able to walk ≥10 m with a walker or support" resulted in the inclusion of patients in the study who would have been classified as functional grade 2 excluded by the Guillain-Barré Syndrome Study Group. The enrollment of less severely affected GBS patients may have masked the effectiveness of TPE. Of greater concern was inadequate matching in the study treatment arms. The TPE group was older and 41% of subjects had diminished compound muscle potentials at baseline. In comparison, the IVIg group was younger with diminished compound muscle potentials in only 27% of patients. Age and diminished compound muscle potentials are the two most reliable negative prognostic factors for response to TPE in GBS. Similar problems with equivalency of the TPE and IVIg groups were also seen in a more recent study by Alshekhlee et al where patients receiving TPE were older with higher complication rates and, not surprisingly, higher mortality rates [20].

The second larger trial by the Plasma Exchange/Sandoglobulin Guillain-Barré Syndrome Trial Group found equivalency between IVIg and TPE in patients across multiple disability parameters, with equivalent low rates of treatment complications and numbers of deaths [3]. While TPE could, in theory, increase the risk of infection and hemorrhage through the removal of immunoglobulins and clotting factors, neither AEs occurred more frequently in the TPE group compared to the IVIg group in this trial [3] or in trials of TPE tested against supportive care.

Table 4 shows AEs associated with TPE and IVIg treatment. The side-effect profiles are similar with regard to severity and frequency of mild and severe reactions. The profiles differ with regard to types of reactions and their underlying pathophysiology.

**Table 4:** Side-effect profiles of intravenous immunoglobulin infusion and therapeutic plasma exchange

**Intravenous Immunoglobulin Infusion **[25-28]	**Therapeutic Plasma Exchange **[21-23]
**Mild (common)**	**Mild (common)**
Fever	Fever
Facial flushing	Paresthesias due to hypocalcemia
Malaise	Hematoma at site of vascular access
Headache	Bleeding at site of vascular access
Chills	Muscle cramping due to hypocalcemia
Myalgia	Nausea
Fatigue	Vomiting
Dyspnea	Pallor
Back Pain	Diaphoresis
Abdominal pain	Hypotension
Nausea	Tachycardia
Vomiting	Urticaria
Diarrhea	Pruritis
Hypotension	Hypofibrinogenemia
Tachycardia	
Urticaria	
Pruritis	
Pseudohyponatremia	
**Severe (uncommon)**	**Severe (uncommon)**
Anaphylaxis	Anaphylaxis
Acute renal failure	Air embolism
Thromboembolic events	Arrhythmia
Stroke	Hemolysis
Myocardial infarction	Thrombocytopenia
Deep venous thrombosis	Transfusion Related Acute Lung Injury
Aseptic meningitis	Pneumothorax due to central line
Progressive neurodegeneration	Hemothorax due to central line
Serum sickness	Tetany
Hemolysis	
Neutropenia	
Transfusion Related Acute Lung Injury	
Uveitis	
Leukocytoclastic vasculitis	
Erythema multiforme	

The frequency of adverse complications associated with TPE have been reported to occur in 4.75% to 36% of procedures [21-23]. The vast majority of reactions are mild, easily treated, and self-limited [21-23]. Severe reactions, defined as those with the potential to be life threatening, have been reported to occur in 0.12% of TPEs [21]. Couriel and Weinstein observed a higher incidence of severe reactions, 6.15%, with all being related to central line placement complications where only 23% of the patients were treated via peripheral access [22]. In contrast, Basic-Jukic et al noted a lower reaction rate in a study where 72% of the TPE procedures were performed with peripheral vascular access [21]. Thus with TPE the frequency of reactions directly attributable to the procedure performed via peripheral access is less than those due to central line placement [22]. While it is frequently assumed that all patients undergoing TPE for neurologic diseases require central line placement, studies demonstrating that a majority (72 to 96%) of patients can satisfactorily undergo TPE using peripheral vascular access suggest otherwise [22,24].

Frequency of adverse effects of IVIg has been reported to occur in 11 to 81% of patients receiving infusions with more recent studies demonstrating reaction rates of 36% to 42% [25,26]. As with TPE, the majority are mild, easily treated, and self-limited. The frequency of severe, life threatening, reactions is rare [25-28].

Reducing unnecessary costs is critical to addressing the growing lack of affordability of health care, now reflected in the fact that 15% of Americans do not have health insurance [29]. Hospitals are motivated to find opportunities to reduce costs without compromising patient outcome, particularly in the inpatient setting where prospective payment arrangements with public- and private-sector insurers reward cost containment. Reimbursement in most instances is defined prospectively and remains fixed regardless of the actual costs of care for an individual patient. Medicare, for example, pays most hospitals a fixed payment amount based on the Diagnosis Related Group (DRG) to which the stay is assigned. Most private insurers pay a fixed per diem rate that is typically independent of how much is spent on procedures, drugs, supply items and other resources.

Since the Plasma Exchange/Sandoglobulin Guillain-Barré Syndrome trial, the presumption of similar costs may have influenced neurologists to use IVIg as a first-line for treatment of GBS rather than TPE. Similarly, the use of IVIg rather than TPE in the management of other neurological conditions (Table 5), including chronic inflammatory demyelinating polyneuropathy and myasthenia gravis may also be affected by this perspective [30-33].

**Table 5:** Neurologic diseases treated with both intravenous immunoglobulin infusion and therapeutic plasma exchange

Neurologic Disease	**Role of intravenous immunoglobulin infusion in treatment **[33]	**Role of therapeutic plasma exchange in treatment **[13]
Acute inflammatory demyelinating polyneuropathy (AIDP, Guillain-Barré syndrome)	Definite	First line therapy with a strong recommendation and high-quality evidence. (Category I, Recommendation grade 1A)
Chronic inflammatory demyelinating polyneuropathy (CIDP)	Definite	First line therapy with a strong recommendation and moderate quality evidence. (Category I, Recommendation grade 1B)
Lambert-Eaton myasthenic syndrome (LEMS)	Probable	Second line therapy with a weak recommendation and low quality or very low quality evidence. (Category II, Recommendation grade 2C)
IgM anti-myelin associated glycoprotein paraprotein associated peripheral neuropathy	Probable	First line therapy with a strong recommendation and low quality or very low quality evidence. (Category I, Recommendation grade 1C)
Myasthenia gravis (MG)	Probable	First line therapy with a strong recommendation and high-quality evidence. (Category I, Recommendation grade 1A)
Multiple sclerosis (MS)	Possible	Second line therapy with a strong recommendation and moderate quality evidence. (Category II, Recommendation grade 1B)
Acute disseminated encephalomyelitis (ADEM)	Possible	Second line therapy with a weak recommendation and moderate quality evidence. (Category II, Recommendation grade 2C)

The average price for IVIg since the Plasma Exchange/Sandoglobulin Guillain-Barré Syndrome trial has more than doubled from approximately $30 per gram to $70.22 as of April 6, 2010 [3]. While the cost of HSA used for volume replacement in TPE is not inconsiderable, it is dwarfed by a 2.2 times higher average IVIg price. Use of less than an average of 2.8 liters of HSA per procedure due to smaller patient plasma volumes further widens this HSA-IVIg cost gap. Other resources required to perform a TPE procedure (e.g. the nurse operator, disposables including the apheresis tubing set, and amortization of the blood processing device) are less than 25% of the cost of a series of five IVIg infusions used to treat a 70 kg adult patient.

Little has been published comparing the costs of IVIg and TPE for GBS. Early studies found TPE to be cost-saving [34]. However, in 1999; Canadian investigators published a cost minimization analysis that may have overstated costs for IVIg and albumin. While the Canadian group found that TPE was more cost effective than IVIg (TPE $6,204/patient versus IVIg $10,165/patient), they may have inadvertently incorporated published "list" prices for IVIg products, producing an averaged cost estimate roughly two-fold higher than the actual U.S. market price for IVIg at that time [35]. An assumed average cost of $90 per 250 ml bottle of HSA was again dramatically higher than the U.S. hospital-level price of HSA at any time over the last decade. This group also applied an hourly nurse labor cost ($15.25) that was substantially lower than 1999 prevailing U.S. hourly wage ($21.38) for registered nurses [36]. Such cost assumptions, coupled with an escalation in prices of IVIg products over the last several years, supports a re-examination and cost analysis of IVIg and TPE in GBS. Another notable exception to the lack of economic analyses is the work by Tsai, Wang and Liu [37]. The authors retrospectively examined 24 patients with GBS who were admitted to the Taipei Veterans General Hospital, 10 of whom were treated with TPE, seven with IVIg, and seven who received supportive treatment and were treated in the study as a control group. They concluded that despite the significantly lower cost for TPE, total costs were lower in the IVIg group [37]. In a response to the study, Buenz raised several critical questions [38]. While the age difference between the two groups was not statistically significant, Buenz suggested that the difference between an 83-year-old and a 45 year-old-patient (statistically equally as probable according to the data presented where the 95% confidence interval for the age difference is -5.12 to 38.5 years) is clinically significant with regard to GBS prognosis, regardless of the therapeutic intervention [38]. Second, the severity of disease and co-morbidities are not adequately described. Since patients receiving IVIg in Taipei must personally pay out-of-pocket expenses for their therapy it is reasonable to question whether wealthier and potentially younger patients with less co-morbidity seen earlier in their disease course may comprise and bias the cohort examined. Finally, the authors state that the incidence of complications is the same while the cost for treatment is higher for the TPE group. One patient in each group had pneumonia. Since there is no reason to assume that the cost of treating pneumonia would be higher in one group or the other, it is assumed that the sole difference in the cost of complications is from a septic patient. The assertion that TPE is more expensive than IVIg is based on this single patient with sepsis where the nature and source of the infection is unreported. While the study provides an important contribution to the literature on TPE and IVIg, it raises concerns that require additional research [38].

In a more recent detailed study of the economic cost of GBS in the U.S., the lifetime health burden in monetary terms for 5,500 GBS patients was examined [39]. Such a study provides information to assess cost-effectiveness of health measures that affect GBS. The estimated annual cost of GBS was $1.7 billion (95% CI, $1.6 to 1.9 billion) including $200 million (14%) in direct medical costs and $1.5 billion (86%) in indirect costs. In our work, we focused solely on assessing the savings in drug costs. At $5,350.00 per course of five treatments multiplied by the number of treatments per year times the number of GBS patients treated at a given hospital, a significant budget impact on pharmacy costs could quickly accrue.

We intentionally did not consider "indirect cost" that hospitals may assign to the physical floor space required to perform a TPE procedure in our analysis. Space valuation is variable between institutions, reflecting differences in revenue and accounting practices. This valuation would be small on a per-procedure basis and therefore would not significantly affect this analysis. In addition, assignment of an indirect cost for physical space is financially relevant only if the treatment of this patient population requires addition of an apheresis medicine treatment unit. If existing apheresis medicine service capacity can absorb those procedures, it is difficult to argue that the hospital has incurred a new "cost."

Our study is limited in three significant ways. First, while AEs attributable to TPE and IVIg occurred with similar frequencies in the 1997 Plasma Exchange/Sandoglobulin Guillain-Barré Syndrome Trial, the nature of those events was different between the two groups. It is therefore possible that the overall costs of treating these AEs will also be dissimilar. This, however, may be of no importance since the complications for both treatments are typically mild and self-limited requiring minimal intervention. Second, we did not consider the influence of either abbreviated TPE or IVIg treatment courses on overall costs. In the Plasma Exchange/Sandoglobulin Guillain-Barré Syndrome Trial, less than 75% of the planned intervention was given to 13.8% of patients in the TPE group versus just 2.3% in the IVIg group. All subjects were included in the outcomes analysis. In addition, alternate replacement fluids and/or a lower percentage of HSA in the replacement fluid could be used, though this is not consistent with published guidelines. If similar under-dosing rates or use of alternate replacement fluids occur in current practice, this would result in further reductions of the average cost of TPE. Finally, our study examines only the direct costs of TPE and IVIg, not the total costs. In order to determine total costs it would be necessary to have data on the types and frequency of adverse effects of the two treatments as well the costs associated with treating these complications. Contemporary data is not available.

## Conclusions

By treating GBS patients with a course of five TPE treatments instead of five IVIg infusions, U.S. hospitals can expect to realize a cost savings of more than $5,680.00. For a given hospital, the magnitude of this cost advantage will be only marginally affected by its average IVIg and albumin acquisition costs and local nursing wage rates. Considering the high cost of IVIg as a maintenance therapy, especially for other conditions like chronic inflammatory demyelinating polyneuropathy and myasthenia gravis, institutions may realize significant savings. Based not only on its efficacy and safety profiles but on its financial considerations as well, TPE should be strongly viewed as first line therapy in the treatment of GBS patients.

## Abbreviations

ADEM: acute disseminated encephalomyelitis; AE: adverse event; AIDP: acute inflammatory demyelinating polyneuropathy; APC: ambulatory payment classification; ASP: average sale price; AWP: average wholesale price; CIDP: chronic inflammatory demyelinating polyneuropathy; CMS: Centers for Medicare and Medicaid Services; CPEP: Clinical Practice Expense Panel; CPT: Current Procedural Terminology; CY: calendar year; DRG: Diagnosis Related Group; g: gram; GBS: Guillian-Barré syndrome; HAS: human serum albumin; IVIg: intravenous immunoglobulin; kg: kilogram; LEMS: Lambert-Eaton myasthenic syndrome; MG: myasthenia gravis; ml: milliliter; MS: multiple sclerosis; OPPS: outpatient prospective payment system; PEAC: Practice Expense Advisory Committee; PICC: peripherally inserted central catheter; Q1: first quarter; RN: registered nurse; RUC: Relative value scale update committee; TPE: therapeutic plasma exchange; US: United States of America

## Declaration of Competing interests

This study was sponsored by CaridianBCT.

Dr. Winters has received funding from CaridianBCT to investigate the use of the Spectra Optia in the collection of hematopoietic progenitor cells for stem cell transplantation. He is currently the president of the American Society for Apheresis and serves as an editorial board member for the *Journal of Clinical Apheresis*.

Dr. Brown reports no competing interests.

Dr. Hazard worked as a paid consultant (ACE Strategic Reimbursement) to CaridianBCT. She is a health economist with over 20 years experience consulting to the pharmaceutical, biotechnology and device industries.

Mr. Chainani worked as a paid consultant (ACE Strategic Reimbursement) to CaridianBCT until December 2009. Since January 2010, he has been an employee of CaridianBCT heading their HEOR, Pricing and Reimbursement Group. He has 30 years of diversified experience in pharmaceutical, biotechnology, device industries and consulting.

Dr Andrzejewski has served in a paid consultant capacity to the Gerson Lehrman Group as a subject matter expert on issues related to blood component therapy including the use of albumin and intravenous IgG products and apheresis medicine applications. He is a Past President of the American Society for Apheresis and serves as an editorial board member for the *Journal of Clinical Apheresis*.

## Authors' contributions

JW and CA conceived of the study, participated in its design, collected institutional data, and helped draft the manuscript. DB participated in the study design, collected institutional data, and helped draft the manuscript. EH and AC collected government data, created the cost minimization model, and helped draft the manuscript. All authors read and approved the final manuscript.

## Pre-publication history

The pre-publication history for this paper can be accessed here:

http://www.biomedcentral.com/1472-6963/11/101/prepub

## Supplementary Material

Additional file 1 **The additional data file consists of a spreadsheet (Excel 2003, Microsoft Corporation, Redmond WA) that contains the model used in this manuscript to perform the cost minimization analysis**. Individual institutions can input their own direct costs and reimbursement amounts from any specific payer and perform the analysis reported in this study in real-time. Click here for file
